# Duration of dual antiplatelet therapy after drug-eluting stent implantation: Meta-analysis of large randomised controlled trials

**DOI:** 10.1038/srep13204

**Published:** 2015-08-17

**Authors:** Man-Fung Tsoi, Ching-Lung Cheung, Tommy Tsang Cheung, Ian Chi-Kei Wong, Cyrus Rustam Kumana, Hung-Fat Tse, Bernard Man-Yung Cheung

**Affiliations:** 1 Division of Clinical Pharmacology and Therapeutics, Department of Medicine, The University of Hong Kong, Pokfulam, Hong Kong, China; 2 Department of Pharmacology and Pharmacy, The University of Hong Kong, Pokfulam, Hong Kong, China; 3 Research Centre of Heart, Brain, Hormone and Healthy Aging, The University of Hong Kong, Pokfulam, Hong Kong, China; 4 Partner State Key Laboratory of Pharmaceutical Biotechnology, The University of Hong Kong, Pokfulam, Hong Kong, China; 5 Centre for Genomic Sciences, The University of Hong Kong, Pokfulam, Hong Kong, China; 6 Division of Cardiology, Department of Medicine, The University of Hong Kong, Pokfulam, Hong Kong, China; 7 Institute of Cardiovascular Science and Medicine, The University of Hong Kong, Pokfulam, Hong Kong, China

## Abstract

Patients receive dual antiplatelet therapy (DAPT) for 6–12 months after drug-eluting stents (DES) implantation. The efficacy and safety of prolonged DAPT has been questioned. Therefore, we performed a meta-analysis on randomised trials comparing different DAPT durations. Literature was searched on trials comparing different DAPT durations. For inclusion, reports must report frequency of cardiovascular and bleeding events. Ten trials were included. Compared to 12 months, DAPT beyond 12 months was associated with fewer myocardial infarctions (OR 0.58 95%CI: 0.40–0.84) and stent thrombosis (OR 0.35 95%CI: 0.20–0.62), but more major bleeds (OR 1.60 95%CI: 1.22–2.11) and all-cause (OR 1.30 95%CI: 1.02–1.66) mortality. There was no significant alteration in risk of stroke (OR 0.93 95%CI: 0.66–1.31) or cardiac (OR 1.12 95%CI: 0.73–1.71) mortality. Compared to less than 12 months DAPT, 12 months DAPT did not reduce risk of myocardial infarction, stent thrombosis, strokes, cardiac or all-cause mortality, but increased the risk of major bleeds (OR 1.60 95%CI: 1.22–2.11). DAPT beyond 12 months reduce risk of myocardial infarction and stent thrombosis, but there is substantial increase in major bleeding risk and all-cause mortality which need to be addressed. DAPT beyond 12 months does not appear to alter the risk of stroke.

Coronary artery disease is one of the leading causes of morbidity and mortality in developed and developing countries. Atheroma in coronary arteries reduces myocardial blood flow, leading to ischaemia and angina. Percutaneous coronary intervention is now widely performed in conjunction with medical therapy to relieve angina and improve exercise tolerance. After balloon angioplasty, implantation of a metallic stent helps to prevent recoil and restenosis. The stents used nowadays are usually coated with a polymer that elutes a drug such as sirolimus, paclitaxel, everolimus or zotarolimus to suppress neointimal hyperplasia. These drug-eluting stents (DES) delay endothelial healing and may increase the risk of stent thrombosis, but this can be reduced by dual antiplatelet therapy (DAPT).

Conventionally, patients receive DAPT for 6–12 months after DES implantation^1^,^2^. The efficacy and safety of prolonged dual antiplatelet therapy (DAPT) has been questioned. In clinical trials comparing different durations of DAPT, divergent results have been observed^3^,^4^,^5^,^6^,^7^,^8^,^9^,^10^,^11^,^12^. In general, DAPT regimes shorter than 12 months have not been found to be detrimental, and have the advantage of fewer episodes of major bleeding^13^,^14^. However, a low percentage of late stent thrombosis remains a challenge. Recently, several clinical trials that examined whether longer periods of DAPT are beneficial have been completed^7^,^9^,^10^,^11^. There is therefore a need to re-examine, in the light of these new trials, the question of whether DAPT for longer than 12 months in patients who have received DES is efficacious and safe compared to DAPT for 12 months and less than 12 months. We used the powerful technique of meta-analysis to determine any reduction in cardiovascular events and any increase in serious adverse events such as bleeding or death.

## Methods

We searched for randomised trials comparing different durations of DAPT (aspirin + P2Y_12_ inhibitor) after DES implantation on 18 November 2014. PubMed, EMBASE, Scopus, Cochrane database of systematic reviews, recent meta-analyses on the subject, recent cardiology conference abstracts and ClinicalTrials.gov were searched using the search term “Dual Antiplatelet therapy”, “Myocardial infarction”, “Stent thrombosis”, “Stroke”, “Drug Eluting Stent” and “Bleeding”. For inclusion, the report had to contain the frequency of cardiovascular and bleeding events. A summary of the search process for the trials is shown in Supplementary Fig. 1. The inclusion criteria were (1) articles or abstracts written in English; (2) participants had to be aged 18 or older; (3) patients had to be randomized to receive different durations of DAPT. Analyses of non-randomized trial subgroups were excluded. Data extraction and assessment of bias were performed by two investigators. The trials selected for inclusion were stratified into three groups according to the durations of DAPT: (1) >12 months DAPT vs. 12 months DAPT; (2) >12 months DAPT vs. <12 months DAPT; and (3) 12 months DAPT vs. <12 months DAPT.

Efficacy outcomes were the frequency of myocardial infraction, stroke and stent thrombosis. The safety outcomes were the rate of cardiac and all-cause mortality, and the frequency of bleeding. The meta-analysis was performed using RevMan (version 5.3.4). Odds ratios and 95% confidence intervals of each trial and combination of trials were calculated using the random effects model. I^2^ statistics were calculated to evaluate heterogeneity among studies. Sensitivity analysis was undertaken to evaluate the effect of the inclusion or exclusion of a trial on the summary odds ratio. Bias in the selection or publication of studies was assessed using funnel plots, Begg’s, Egger’s and trim-and-fill tests. A P-value of <0.05 was taken to indicate statistical significance. We followed the PRISMA Statement on the reporting of meta-analysis.

We calculated the number-needed-to-treat (NNT) to prevent one stent thrombosis and the number-needed-to-harm (NNH) for major bleed in the DAPT study as the reciprocal of the change in absolute risk, which was the difference in proportion of patients with these events in the two arms of the study^15^. These are expressed as NNT or NNH per year as the length of follow-up was 18 months.

## Results

Ten trials were included in the meta-analysis^3^,^4^,^5^,^6^,^7^,^8^,^9^,^10^,^11^,^12^. A summary of their characteristics and the risk of bias is shown in Table 1, Supplementary Table S1 and Supplementary Table S2.

**Figure 1:**
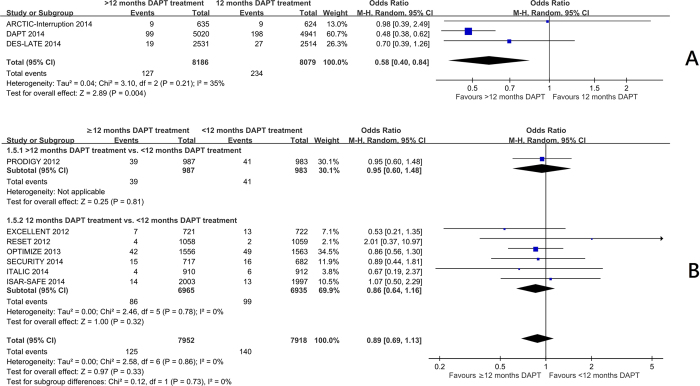
(**A**) The effect of prolonging the duration of dual antiplatelet therapy to more than 12 months on frequency of myocardial infarction in patients after drug-eluting stent implantation. (**B**) The effect of shortening the duration of dual antiplatelet therapy to less than 12 months on the frequency of myocardial infarction in patients after drug-eluting stent implantation.

**Figure 2:**
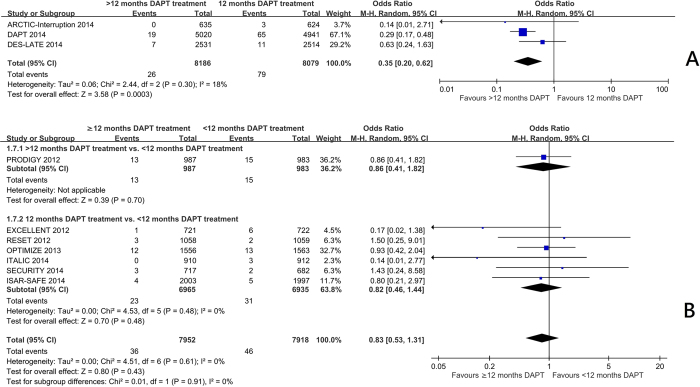
(**A**) The effect of prolonging the duration of dual antiplatelet therapy to more than 12 months on frequency of definite or probable stent thrombosis in patients after drug-eluting stent implantation. (**B**) The effect of shortening the duration of dual antiplatelet therapy to less than 12 months on the frequency of definite or probable stent thrombosis in patients after drug-eluting stent implantation.

**Figure 3:**
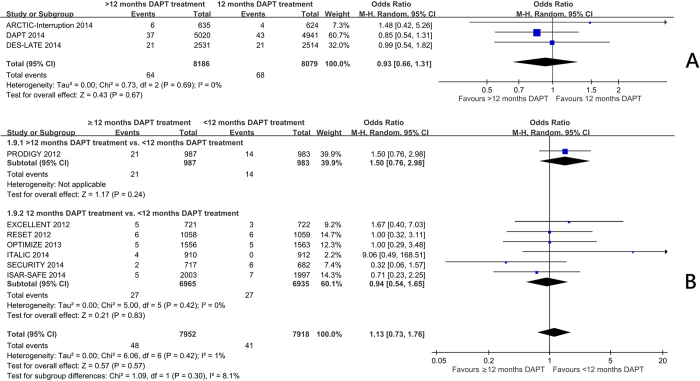
(**A**) The effect of prolonging the duration of dual antiplatelet therapy to more than 12 months on frequency of stroke in patients after drug-eluting stent implantation. (**B**) The effect of shortening the duration of dual antiplatelet therapy to less than 12 months on frequency of stroke in patients after drug-eluting stent implantation.

**Figure 4:**
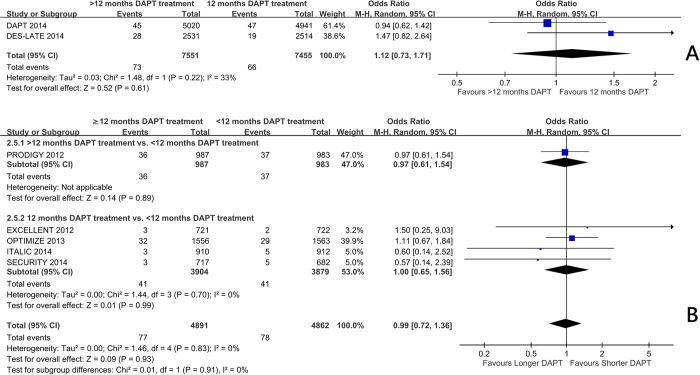
(**A**) The effect of prolonging the duration of dual antiplatelet therapy to more than 12 months on cardiac death rate in patients after drug-eluting stent implantation. (**B**) The effect of shortening the duration of dual antiplatelet therapy to less than 12 months on cardiac death rate in patients after drug-eluting stent implantation.

**Figure 5:**
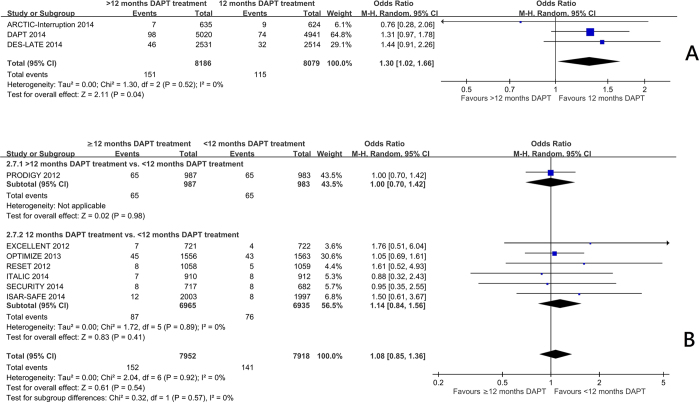
(**A**) The effect of prolonging the duration of dual antiplatelet therapy to more than 12 months on all-cause mortality rate in patients after drug-eluting stent implantation. (**B**) The effect of shortening the duration of dual antiplatelet therapy to less than 12 months on all-cause mortality rate in patients after drug-eluting stent implantation.

**Figure 6:**
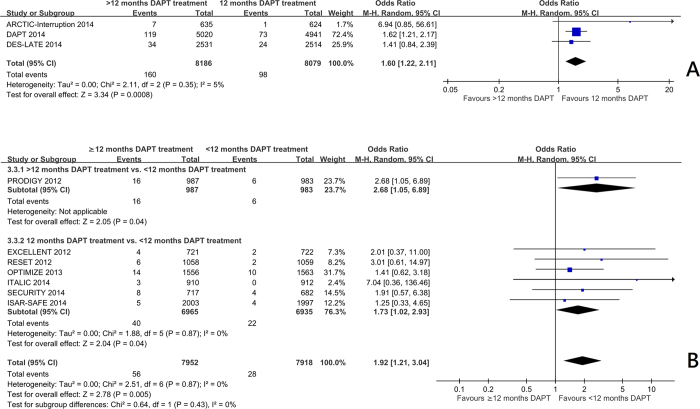
(**A**) The effect of prolonging the duration of dual antiplatelet therapy to more than 12 months on frequency of major bleeding in patients after drug-eluting stent implantation. (**B**) The effect of shortening the duration of dual antiplatelet therapy to less than 12 months on the frequency of major bleeding in patients after drug-eluting stent implantation.

Trials comparing 12 months with beyond 12 months of DAPT^7^,^10^,^11^ showed a significant reduction in myocardial infarction frequency (OR 0.58 95%CI: 0.40 to 0.84, p = 0.004) (Fig. 1A) and stent thrombosis (OR 0.35 95%CI: 0.20 to 0.62, p = 0.0003) (Fig. 2A). There was no significant increase in the risk of stroke (OR 0.93 95%CI: 0.66 to 1.31) (Fig. 3A), or cardiac death (OR 1.12 95%CI: 0.73 to 1.71) (Fig. 4A), but the risk of all-cause mortality (OR 1.30 95%CI 1.02–1.66) (Fig. 5A) and major bleeding were increased (OR 1.60 95%CI: 1.22 to 2.11) (Fig. 6A).

Trials comparing less than 12 months of DAPT with beyond 12 months of DAPT^5^ and 12 months of DAPT with less than 12 months of DAPT^3^,^4^,^6^,^8^,^9^,^12^ showed no significant increase in the risk of myocardial infarction (Fig. 1B), definite or probable stent thrombosis (Fig. 2B), stroke (Fig. 3B), cardiac death (Fig. 4B) or all-cause mortality (Fig. 5B), but the risk of major bleeding were increased (Fig. 6B).

There was no significant heterogeneity among the trials on stroke, all-cause mortality, or major bleeding (I^2^ = 0) in three groups. There was no significant heterogeneity among the trials among the trials in the effect on myocardial infarction, stent thrombosis and cardiac death in trials comparing 12 months and <12 months of DAPT, and trials comparing >12 months and <12 months of DAPT. There was heterogeneity among the trials in the effect on myocardial infarction, stent thrombosis and cardiac death in trials comparing 12 months and >12 months of DAPT (I^2^ = 38%, 18% and 33% respectively, p = 0.21, 0.30, 0.22 respectively) (Figs 1A, 2A and 4A). Hence, the trials comparing 12 months and >12 months of DAPT were analysed in sensitivity analysis.

The sensitivity analysis is summarised in Supplementary Table S3, S4 and S5, which shows the effect of including and excluding each trial on the summary OR and I^2^. The heterogeneity in the risk of myocardial infarction (I^2^ = 35%, p = 0.21) was due to DAPT study; omitting DAPT study reduced I^2^ to 0% and resulted in a non-significant OR. The heterogeneity in the risk of stent thrombosis (I^2^ = 18%, p = 0.30) was due to DAPT and DES-LATE study; omitting either one of the studies reduced I^2^ to 0%. Omitting DAPT study resulted in a non-significant OR while omitting DES-LATE study did not result in non-significant OR. The heterogeneity in the risk of cardiac death (I^2^ = 33%, p = 0.22) was due to DAPT and DES-LATE study; omitting either one of the studies reduced I^2^ to 0 and resulted in a non-significant OR.

Funnel plots of the standard error of the log OR against log OR (Supplementary Figs S2A–S7B) did not show any significant bias (Supplementary Table S6), except for major bleeding (Supplementary Fig. S7B). The two extreme outliers correspond to the ITALIC study. The large ORs were due to the very small number of major bleeds among those on placebo (1 and 0 respectively) (Fig. 6).

In the DAPT study, the NNT/year to prevent one stent thrombosis was 160 (95% CI: 116 to 260) and the NNH/year for major bleed was 167 (95%CI: 105 to 423).

## Discussion

Previous meta-analyses of trials comparing short (≤6 months) and long duration (≥12 months) DAPT have not shown any significant benefits for longer duration treatment; instead, there was significantly increased frequency of major bleeding^13^,^16^. Therefore, many believed that a duration of DAPT shorter than 12 months is not significantly inferior in preventing stent thrombosis and myocardial infarction, but it would reduce exposure of patients to intense antiplatelet treatment with the resultant risk of major bleeds. Relatively small trials exploring the possible benefits of extending DAPT to longer than 12 months did not have sufficient power to show beneficial effects^5^,^7^,^9^,^10^. The DAPT study was a large trial that showed a significant reduction in the number of patient stent thrombosis and myocardial infarction. Thus, it confirms the trend towards reduction in these events in the previous trials. However, not only was there an expected increase in major bleeding, there was also a trend towards increased mortality^11^. Although a concurrent meta-analysis showed that DAPT in general did not cause excess mortality, the results of the DAPT study were surprising and required confirmation^17^. The present meta-analysis is ground-breaking in establishing that DAPT longer than 12 months after DES implantation markedly reduces myocardial infarction and stent thrombosis. This finding provides a rationale for considering prolonged DAPT in these patients.

However, prolonged DAPT is not without risk. In the DAPT study, for every stent thrombosis prevented, nearly one major bleed occurred^11^. Thus, clinical judgment is necessary to weigh up the benefits and risks in the individual patient, and explain them to the patient in a way that can be understood. In this regard, we do not have the equivalent of validated risk scores such as CHA_2_DS_2_-VASc and HAS-BLED that facilitate decisions to anticoagulate patients with atrial fibrillation^14^,^18^. Besides intensive antiplatelet therapy, we do not have other effective means of preventing stent thrombosis in procedurally successful DES implantations, though there may be ways of reducing the bleeding risk. In the setting of prolonged DAPT, it remains to be proven whether careful patient selection based on medical history, helicobacter eradication, and prophylaxis with proton pump inhibitor can minimise this risk. The physician should now endeavour to do this before denying patients DAPT because of perceived bleeding risks.

As regards mortality, although a recent meta-analysis on DAPT found no significant increase in mortality with prolonged treatment^17^, that meta-analysis did not include ISAR-SAFE or ITALIC. A meta-analysis that compared shorter and longer DAPT found a non-significant increase in all-cause mortality^19^. A meta-analysis using individual patient data did not show any significant increase in all-cause mortality, but that might have been due to the availability of individual patient data in only four trials^20^. A more recent meta-analysis showed a 22% increase in all-cause mortality and a 49% increased rate of non-cardiac mortality associated with DAPT beyond 12 months^21^. Our meta-analysis showed similar findings. Until we have more data on the safety of DAPT beyond 12 months, the large reduction in myocardial infarction brought about by DAPT beyond 12 months has to be weighed against an increased risk of major bleeds and mortality. Whether or not major bleeds become life-threatening is influenced by the availability and timeliness of the emergency medical care, which can vary from place to place.

The neutral effect of DAPT on stroke rate is interesting. Antiplatelet therapy is expected to reduce thrombotic strokes but may increase the rate of haemorraghic strokes or haemorrhagic transformation of an ischaemic stroke. More studies are needed to explore this issue. At least, we can conclude that DAPT does not increase the risk of stroke.

Before implementing long-term DAPT for DES patients, the limitations of clinical trial evidence must not be overlooked. Trials recruit voluntary patients who may be more compliant with treatment. Inclusion and exclusion criteria create trial populations that may not be completely representative of patients in the real world. Patients with certain co-morbidities and those at high risk of bleeding are excluded. Patients with major bleeding in first year of DAPT therapy were excluded in DAPT, ARCTIC-Interruption and DES-LATE study. Thus, DAPT was only continued beyond one year in those who could tolerate it in the first year. Not all patients would be suitable for DAPT beyond 12 months.

There were also differences among the trials with respect to the types of DES used and the definition of myocardial infarction and major bleeding. Different types of DES, especially newer stents, may differ in the optimal duration of DAPT. Second generation DESs are known to be as effective as first generation DESs, but with a lower risk of stent thrombosis^22^. First generation DESs were used in some of the patients in DES-LATE, ARCTIC-Interruption and the DAPT study, but second generation DESs are now the main types of stents in clinical use. As the rate of stent thrombosis is low with second generation DES, it would be difficult to do a trial with enough stent thrombosis events to analyse; and with a very low rate of stent thrombosis, it may not be clinically important to reduce this rate further. However, optimal coherence tomography shows that incomplete stent apposition is not uncommon at 12 months after implantation of everolimus-eluting stents, a second generation DES^23^. Even with a biodegradable polymer stent, stent thrombosis still occurs after one year^24^. Therefore, advances in stent technology have reduced stent thrombosis but have not eliminated it. In patients with coronary artery atherosclerosis, the thrombus causing myocardial infarction need not be at the site of previous stenting. DAPT may therefore have benefits beyond its effect on reducing stent thrombosis.

Definitions of major and minor bleeding varied among the trials; different criteria based on clinical symptoms, laboratory parameters, or both, were used^25^. Although the number of major bleeds in the trials is open to interpretation, there is little doubt that DAPT carries a higher risk of bleeding.

It should also be remembered that myocardial infarctions in clinical trials were often diagnosed on the basis of rises in cardiac troponin or creatine kinase^26^. These small infarcts might not result in severe clinical symptoms or poor prognosis. The prevention of small infarcts may not be a sufficient justification for a treatment that increases the risk of major bleeding and death.

In conclusion, this meta-analysis of randomised trials comparing different durations of DAPT after DES implantation shows benefits in extending DAPT beyond 12 months, in terms of a lower frequency of myocardial infarction and stent thrombosis. There is no significant decrease in cardiac mortality and stroke, but there is an increase in major bleeds and all-cause mortality. DAPT for 12 months or for a shorter duration do not differ with respect to efficacy or safety endpoints. Physicians can consider continuing DAPT beyond 12 months after weighing up the benefits and risks, informing patients fully of the increased risk of major bleeding, and taking steps to minimise this risk.

## Additional Information

**How to cite this article**: Tsoi, M.-F. *et al*. Duration of dual antiplatelet therapy after drug-eluting stent implantation: Meta-analysis of large randomised controlled trials. *Sci. Rep*. **5**, 13204; doi: 10.1038/srep13204 (2015).

## Supplementary Material

Supplementary Information

## Figures and Tables

**Table 1:** Summary for trial design for studies included in meta-analysis.

Study	Number of participants	Intervention	Type of Stent used	Treatment durations	Primary endpoint(s)	Bleeding criteria
RESET 2012^4^	12-month group: 1058 3-month group: 1059	12 months clopidorgrel + aspirin vs 3 months clopidorgrel + aspirin	3-month group: ZES 12-month group: SES, EES, ZES (early gen.)	12 months vs 3 months	Incidence rate of cardiovascular death, MI, ST, TVR, or bleeding) at 1 year	TIMI
EXCELLENT 2012^3^	12-month group: 721 6-month group: 722	12 months clopidorgrel + aspirin vs 6 months clopidorgrel + aspirin	SES and EES	12 months vs 6 months	Composite of cardiac death, MI or major bleeding during month 0–12	TIMI
PRODIGY 2012^5^	24-month group: 987 6-month group: 983	24 months clopidorgrel + aspirin vs 6 months clopidorgrel + aspirin	EES (both early and new generation), PES and BMS#4	24 months vs 6 months	Incidence rate of death of any cause, non-fatal MI or cerebrovascular accident at month 24	TIMI
OPTIMIZE 2013^6^	12-month group: 1563 3-month group: 1556	12 months clopidorgrel + aspirin vs 3 months clopidorgrel + aspirin	ZES	12 months vs 3 months	Composite of all-cause death, MI, ST, stroke, major bleeding at month 12	REPLACE-2, GUSTO
DES-LATE 2014^10^	Continue DAPT: 2531 Discontinue DAPT: 2514	12 months aspirin vs 12 months aspirin + clopidogrel[Fn t1-fn1]	SES, PES, ZES, EES and other DES	24 months vs 12 months#1	Composite of death due to cardiac death, MI and stroke at month 24 after randomization	TIMI
ARCTIC-Interruption 2014^7^	Continue DAPT: 635 Discontinue DAPT: 641	12 months Thienopyridine + aspirin vs 12 months aspirin[Fn t1-fn1]	SES, PES, ZES, EES	18–30 months vs 12 months	Composite of all-cause mortality, MI, stroke, ST, urgent Coronary revascularization or transient ischemic attack at month 30	STEEPLE
SECURITY 2014^8^	6-month group: 682 12-month group: 717	12 months aspirin + 6 months clopidogrel vs 12 months clopidogrel	ZES, EES, Nobori stent, Biomatrix stent, Promus stent	12 months vs 6 months	Composite of cardiac death, MI, stroke, ST, type 3 or 5 bleeding as defined in the criteria at month 12	BARC
ISAR-SAFE 2014^12^	12-month group: 2003 6-month group: 1997	Antiplatelet drug (not specified in the presentation) + 6 months clopidogrel followed by 6 months placebo vs 12 months clopidogrel	SES, PES, ZES, EES, BES, Bioresorbable EES, BMS, drug-coated balloon and plain balloon angioplasty#3	12 months vs 6 months	Composite of death, MI, ST, stroke, major bleeding at month 15	TIMI
ITALIC 2014^9^	24-month group: 910 6-month group: 912	24 months aspirin + 24 months vs 6 months clopidogrel, prasugrel or tricagrlor	ZES	24 months vs 6 months#5	Composite of death, MI, emergency TVR at month 12	TIMI
DAPT 2014^11^	30-month group: 4710 12-month group: 4649	30 months aspirin + 30 months Thienopyridine vs 12 months Thienopyridine followed by 18 months placebo	SES, ZES, PES, BMS#2	30 months vs 12 months	No. of patients with ST and major cardiovascular and cerebrovascular events (including stroke and MI) at month 30	BARC, GUSTO

Abbreviations used in table 1, ST: Stent thrombosis; MI: myocardial infraction; TVR: target vessel revascularization; DES: Drug-eluting stent; gen.: generation; BES: Biolimus-eluting stent; BMS: Bare metal stent; EES: Everolimus-eluting stent; ZES: Zotarolimus-eluting stent; PES: paclitaxel-eluting stent; SES: sirolimus-eluting stent.

^*^Patients in ARCTIC-Interruption and DES-LATE Study had previous DAPT. DES-LATE study had cumulative data for primary and secondary outcomes in the research article supplement.

^#1^DES-LATE study allowed patient enrolment with 1 year or longer after percutaneous coronary intervention.

^#2^DAPT study allowed enrolment of patients with BMS but they were not included in statistical analysis.

^#3^In ISAR-SAFE study, the number of patients who received BMS, drug-coated balloon and plain balloon angioplasty were 8 (0.4%), 8 (0.4%) and 2 (0.1%) for 6-month DAPT; 6 (0.3%), 8 (0.4%) and 2 (0.1%) for 12-month DAPT respectively.

^#4^In PRODIGY study, the number of patients who received BMS were 246 (24.9%) for 6-month DAPT; 246 (25.0%) for 24-month DAPT respectively.

^#5^First year data of ITALIC study has been published.
