# Supplementary material for: Duration of dual antiplatelet therapy after drug-eluting stent implantation: Meta-analysis of large randomised controlled trials
Source: Sci Rep. 2015 Aug 17;5:13204. doi: 10.1038/srep13204 (PMC4538605; doi:10.1038/srep13204)
Supplement: Supplementary Information [file srep13204-s1.pdf]

Supplementary Information for Duration of dual antiplatelet therapy after drug-eluting stent implantation: Meta-analysis of large randomised controlled trials

Title of manuscript:

Duration of dual antiplatelet therapy after drug-eluting stent implantation: Meta-analysis of large randomised controlled trials

Man-Fung Tsoi[1], Ching-Lung Cheung[1,2,3,4,5], Tommy Tsang Cheung[1,3], Ian Chi-Kei Wong[2,3,4], Cyrus Rustam Kumana[1,7], Hung-Fat Tse[3,6,7], Bernard Man-Yung Cheung[1,3,4,7]

[1]Division of Clinical Pharmacology and Therapeutics, Department of Medicine

[2]Department of Pharmacology and Pharmacy

[3]Research Centre of Heart, Brain, Hormone and Healthy Aging

[4]Partner State Key Laboratory of Pharmaceutical Biotechnology

[5]Centre for Genomic Sciences

[6]Division of Cardiology, Department of Medicine

[7]Institute of Cardiovascular Science and Medicine

The University of Hong Kong, Pokfulam, Hong Kong, China

Table of Content

Content	Page
Supplementary Table S1. Summary for blinding, randomization and placebo control of studies included in meta-analysis	2-3
Supplementary Table S2. Risk of Bias Reporting	4
Supplementary Table S3. Sensitivity analysis of the effect of prolonging the duration of dual antiplatelet therapy to more than 12 months on frequency of myocardial infarction in patients after drug-eluting stent implantation	5
Supplementary Table S4. Sensitivity analysis of the effect of prolonging the duration of dual antiplatelet therapy to more than 12 months on frequency of definite or probable stent thrombosis in patients after drug-eluting stent implantation	6
Supplementary Table S5. Sensitivity analysis of meta-analysis the effect of prolonging the duration of dual antiplatelet therapy to more than 12 months on cardiac death rate in patients after drug-eluting stent implantation	7
Supplementary Table S6. Analysis of funnel plots in supplementary figures S2A to S7B to assess publication bias	8
Captions to supplementary figures	9-10

Supplementary table S1. Summary for blinding, randomization and placebo control of studies included in meta-analysis.

Study	Blinding	Randomization assignment	Presence of Placebo	Time of randomization with respect to index DES procedure
RESET[4]	Open-labelled	Assignments were made by an IWRS into ratio of 1:1 based on randomization scheme and stratified by study sites and clinical or lesion characteristics	No placebo control	Before DES implantation
EXCELLENT[3]	Open-labelled	Assignments were made by a Web-based response system.	No placebo control	Before DES implantation
PRODIGY[5]	Open-labelled	Both randomizations for type of stent implantation and DAPT duration were carried out with a computer-generated sequence produced in coordinating center with random block size of 4, 8 and 12.<br><br>Stent Randomization<br>Assignments were made according to randomization scheme into 1:1:1:1 ratio as stratified by study site.<br><br>Randomization of DAPT duration:<br>Assignments were made via a sealed envelope into 1:1 ratio.	No placebo control	Stent randomization:<br>Before DES implantation<br><br>Randomization of DAPT duration:<br>30 +/- 5 days after DES implantation.
OPTIMIZE[6]	Open-labelled	Assignments were made by a dedicated web-based system in blocks into 1:1 ratio and stratified by study sites.	No placebo control	Before DES implantation
DES-LATE[10]	Open-labelled	Assignments were made according to a pre-established, computer generated scheme based on site and type of drug eluting stent.	No placebo control	12-18 months after DES implantation
ARCTIC-Interruption[7]	Open-labelled	Assignments were made by an IVRS into ratio 1:1 ratio and stratified by study site.	No placebo control	1 year after DES implantation
SECURITY[8]	Open-labelled	Assignments were made by electronic case report form into ratio of 1:1 and stratified by study site in block of 4.	No placebo control	Within 1 day after DES implantation
ISAR-SAFE[12]	Double-blinded	Not reported	With placebo control	6 months after DES implantation
ITALIC[9]	Open-labelled	Centralized assignments were made by an IWRS into ratio of 1:1.	No placebo control	6 months after DES implantation

DAPT[11]	Double-blinded	Assignment was made by central IVRS except use of their own IVRS in Boston Scientific Liberte Study. Randomizations were stratified by study site, type of stent implanted (bare metal stent vs. DES),	With placebo control	360 +/- 30 days after DES implantation

Abbreviations used in Supplementary table S1, DAPT: Dual Anti-platelet therapy; DES: Drug-eluting stent; IVRS: Interactive Voice Response System; IWRS: Interactive web-based system

Supplementary table S2. Risk of Bias Reporting.

Study	Sequence generation	Allocation concealment	Blinding of participants and personnel	Blinding of outcome	Incomplete outcome data	Free from other bias
Group I. >12 months treatment vs 12 months treatment						
ARCTIC-Interruption 2014[7]	Low	Low	Unclear	Low	Low	Low
DAPT 2014[11]	Low	Low	Low	Low	Low	Low
DES-LATE 2014[10]	Low	Low	Unclear	Low	Low	Low
Group II. >12 months treatment vs <12 months treatment						
PRODIGY 2012[5]	Low	Low	Unclear	Low	Low	Low
Group III. 12 months treatment vs <12 months treatment						
EXCELLENT 2012[3]	Low	Low	Unclear	Low	Low	Low
RESET 2012[4]	Low	Low	Unclear	Low	Low	Low
OPTIMIZE 2013[6]	Low	Unclear	Unclear	Low	Low	Low
ISAR-SAFE 2014[12]	Low	Unclear	Low	Low	Low	Low
ITALIC 2014[9]	Low	Low	Low	Low	Low	Low
SECURITY 2014[8]	Low	Low	Unclear	Low	Low	Low

Each domain of risk was assigned “Low” for low risk, “Unclear” for unclear risk and “High” for high risk

Supplementary Table S3. Sensitivity analysis of the effect of prolonging the duration of dual antiplatelet therapy to more than 12 months on frequency of myocardial infarction in patients after drug-eluting stent implantation.

Study	Before removing study					After removing study				
	OR	p	I^2	Chi^2	P	OR	p	I^2	Chi^2	P
ARCTIC-Interruption 2014	0.58	0.004	35%	3.10	0.21	0.52	<0.0001	22%	1.28	0.26
DAPT 2014	0.58	0.004	35%	3.10	0.21	0.77	0.30	0%	0.37	0.54
DES-LATE 2014	0.58	0.004	35%	3.10	0.21	0.59	0.11	52%	2.11	0.15

Supplementary Table S4. Sensitivity analysis of the effect of prolonging the duration of dual antiplatelet therapy to more than 12 months on frequency of definite or probable stent thrombosis in patients after drug-eluting stent implantation

.

Study	Before removing study					After removing study				
	OR	p	I^2	Chi^2	P	OR	p	I^2	Chi^2	P
ARCTIC-Interruption 2014	0.35	0.0003	18%	2.44	0.30	0.38	0.01	52	2.09	0.15
DAPT 2014	0.35	0.0003	18%	2.44	0.30	0.55	0.19	0	0.92	0.34
DES-LATE 2014	0.35	0.0003	18%	2.44	0.30	0.28	<0.00001	0	0.22	0.64

Supplementary Table S5. Sensitivity analysis of the effect of prolonging the duration of dual antiplatelet therapy to more than 12 months on cardiac death rate in patients after drug-eluting stent implantation

Study	Before removing study					After removing study				
	OR	p	I^2	Chi^2	P	OR	p	I^2	Chi^2	P
DAPT 2014	1.12	0.61	33%	1.48	0.22	1.47	0.20	Not Applicable		
DES-LATE 2014	1.12	0.61	33%	1.48	0.22	0.94	0.77			

.

Supplementary Table S7. Analysis of funnel plots in supplementary figures 2 to 6 to assess publication bias.

Suppl. Figure	Begg's rank correlation test		Egger's regression intercept	Trim and fill		
				Before trimming	After trimming	
	τ without continuity correction	p-value	p-value	Observed Point estimate (LL-UL)	Trimming direction	Adjusted Point estimate (LL-UL)
S2A	1.000	0.117	0.00971	0.583 (0.404-0.840)	Left	0.482 (0.338-0.687)
S2B	0.143	0.652	0.924	0.886 (0.693-1.13)	Not Applicable	0.886 (0.693-1.13)
S3A	0.333	0.602	0.941	0.350 (0.198-0.620)	Right	0.343 (0.221-0.533)
S3B	-0.238	0.453	0.291	0.833 (0.531-1.31)	Not Applicable	0.833 (0.531-1.31)
S4A	1.000	0.117	0.095	0.928 (0.658-1.31)	Left	0.846 (0.632-1.13)
S4B	0.048	0.881	0.958	1.13 (0.733-1.76)	Not Applicable	1.13 (0.733-1.76)
S5A	Not Applicable*					
S5B	0.067	0.851	0.931	1.01 (0.737-1.38)	Not Applicable	1.01 (0.737-1.38)
S6A	-0.333	0.602	0.510	1.31 (1.03-1.68)	Right	1.36 (1.07-1.73)
S6B	0.429	0.176	0.159	1.05 (0.851-1.36)	Left	1.06 (0.839-1.33)
S7A	0.333	0.602	0.475	1.60 (1.22-2.11)	Left	1.54 (1.04-2.28)
S7B	0.429	0.176	0.204	1.92 (1.21-3.04)	Left	1.78 (1.15-2.75)

Abbreviations used in Supplementary table 5: LL: Lower limit; Suppl. Figure: Supplementary Figure; UL: Upper limit

*Assessment of publication bias for meta-analysis of prolonged dual antiplatelet therapy on cardiac death rate in patients after drug-eluting stent implantation could not be conducted due to number of studies was less than 3.

Captions to Supplementary figures

Supplementary figure S1. Flow diagram of scientific literature search and study selection

Supplementary Figure S2A. Funnel plot showing publication bias for the effect of prolonging the duration of dual antiplatelet therapy to more than 12 months on frequency of myocardial infarction in patients after drug-eluting stent implantation

Supplementary Figure S2B. Funnel plot showing publication bias for the effect of shortening the duration of dual antiplatelet therapy to less than 12 months on frequency of myocardial infarction in patients after drug-eluting stent implantation

Supplementary Figure S3A. Funnel plot showing publication bias for the effect of prolonging the duration of dual antiplatelet therapy to more than 12 months on frequency of definite or probable stent thrombosis in patients after drug-eluting stent implantation

Supplementary Figure S3B. Funnel plot showing publication bias for the effect of shortening the duration of dual antiplatelet therapy to less than 12 months on frequency of definite or probable stent thrombosis in patients after drug-eluting stent implantation

Supplementary Figure S4A. Funnel plot showing publication bias for the effect of prolonging the duration of dual antiplatelet therapy to more than 12 months on frequency of stroke in patients after drug-eluting stent implantation

Supplementary Figure S4B. Funnel plot showing publication bias for the effect of shortening the duration of dual antiplatelet therapy to less than 12 months on frequency of stroke in patients after drug-eluting stent implantation

Supplementary Figure S5A. Funnel plot showing publication bias for the effect of prolonging the duration of dual antiplatelet therapy to more than 12 months on cardiac death rate in patients after drug-eluting stent implantation

Supplementary Figure S5B. Funnel plot showing publication bias for the effect of shortening the duration of dual antiplatelet therapy to less than 12 months on cardiac death rate in patients after drug-eluting stent implantation

Supplementary Figure S6A. Funnel plot showing publication bias for the effect of prolonging the duration of dual antiplatelet therapy to more than 12 months on all-cause mortality rate in patients after drug-eluting stent implantation

Supplementary Figure S6B. Funnel plot showing publication bias for the effect of shortening the duration of dual antiplatelet therapy to less than 12 months on all-cause mortality rate in patients after drug-eluting stent implantation

Supplementary Figure S7A. Funnel plot showing publication bias for the effect of prolonging the duration of dual antiplatelet therapy to more than 12 months on frequency of major bleeding in patients after drug-eluting stent implantation

Supplementary Figure S7B. Funnel plot showing publication bias for the effect of shortening the duration of dual antiplatelet therapy to less than 12 months on frequency of major bleeding in patients after drug-eluting stent implantation

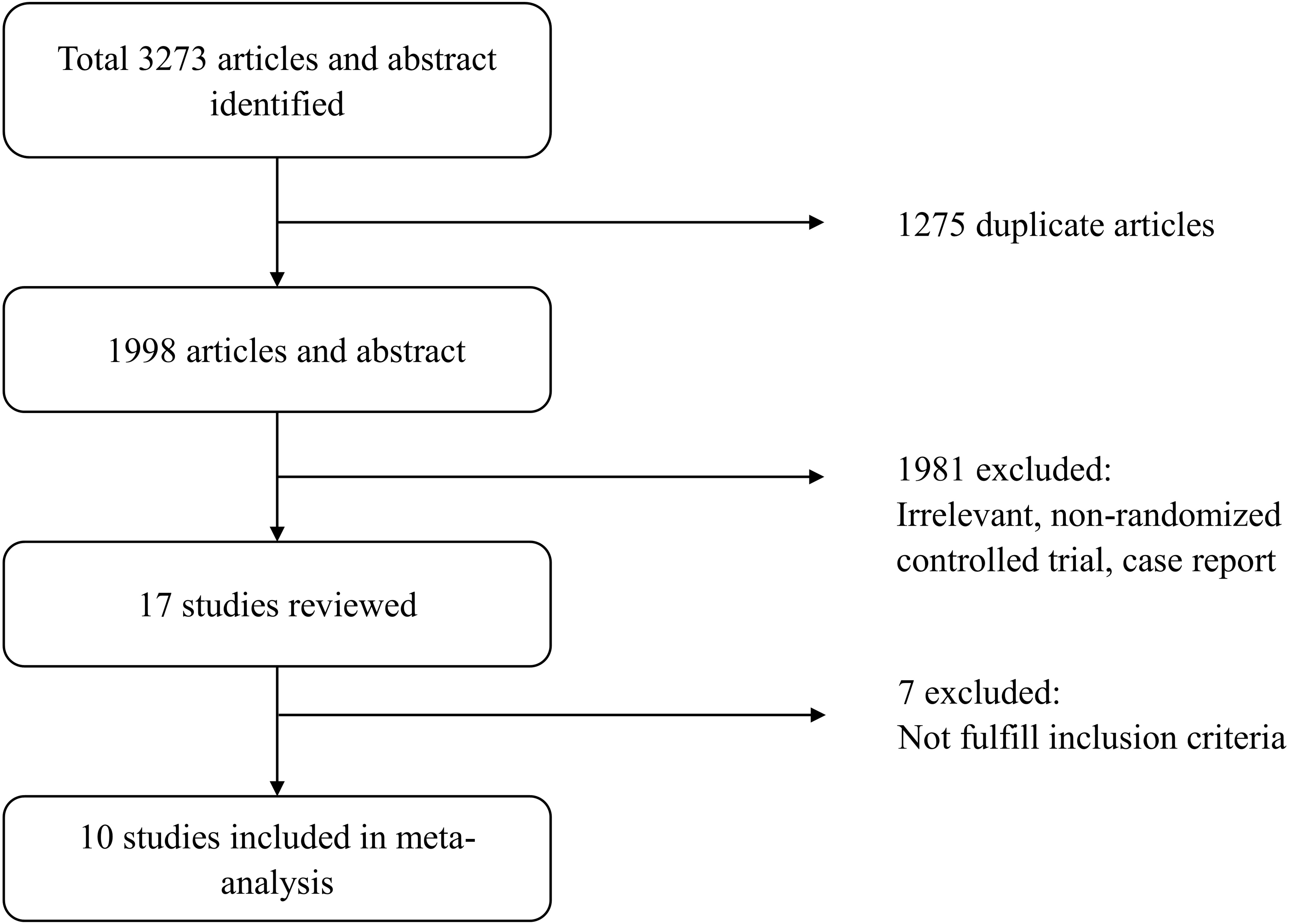
Total 3273 articles and abstract identified
1275 duplicate articles
1998 articles and abstract
1981 excluded:
Irrelevant, non-randomized controlled trial, case report
17 studies reviewed
7 excluded:
Not fulfill inclusion criteria
10 studies included in meta-analysis

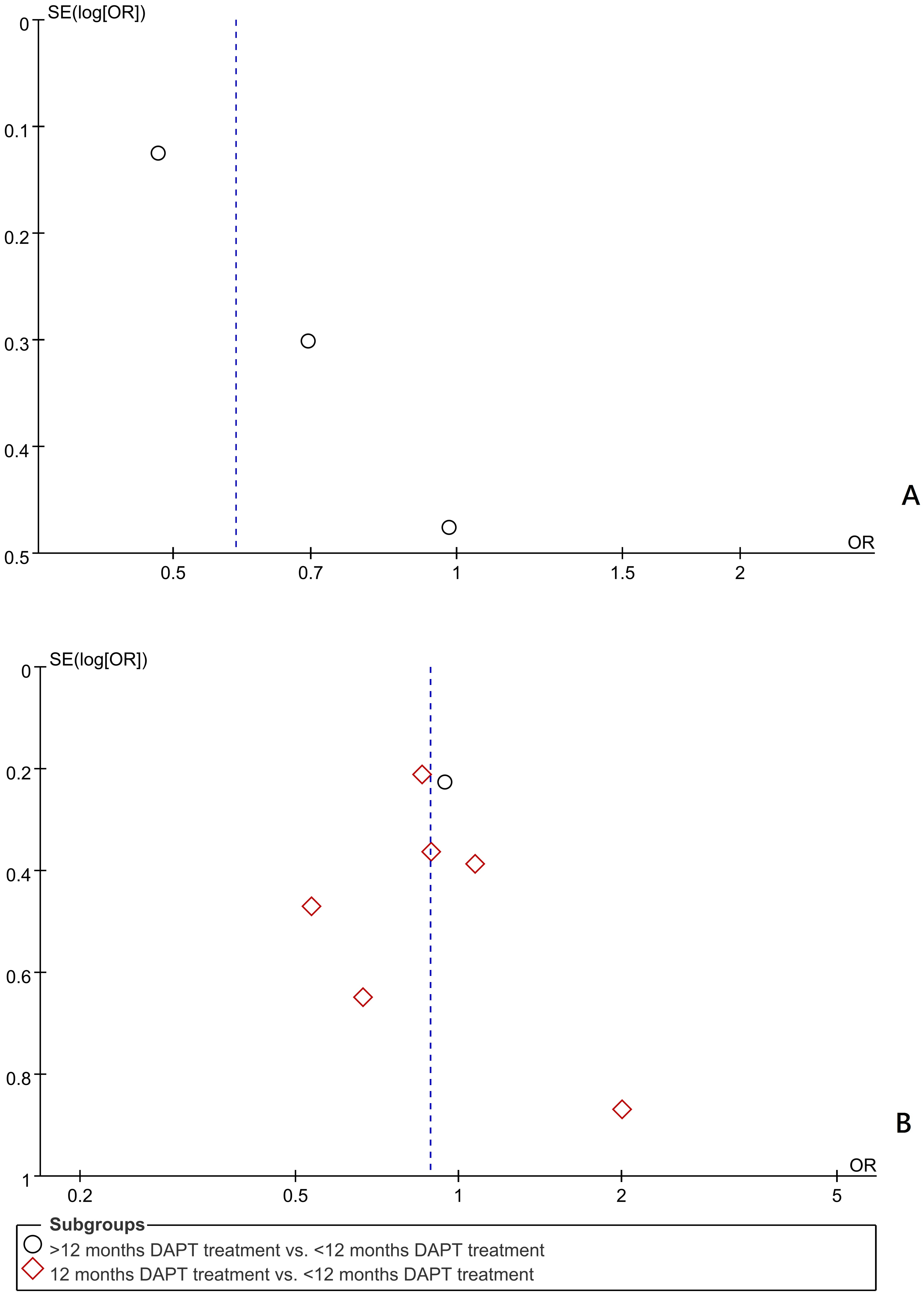

SE(log[OR])
0
0.1
0.2
0.3
0.4
0.5
OR
0.5
0.7
1
1.5
2
A
SE(log[OR])
0
0.2
0.4
0.6
0.8
1
OR
0.2
0.5
1
2
5
B
Subgroups
>12 months DAPT treatment vs. <12 months DAPT treatment
12 months DAPT treatment vs. <12 months DAPT treatment

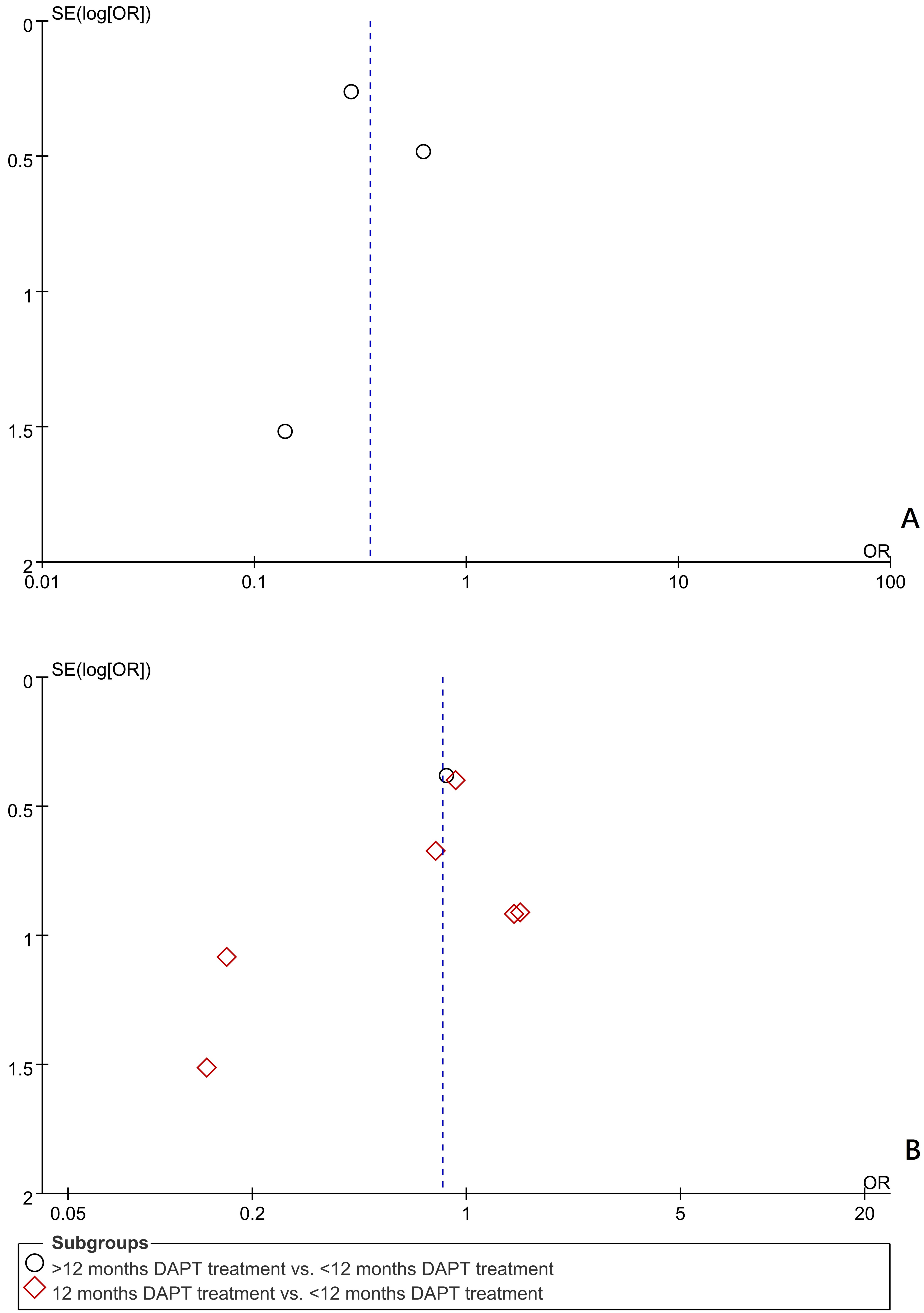
SE(log[OR])
0
0.5
1
1.5
2
A
OR
0.01
0.1
1
10
100
SE(log[OR])
0
0.5
1
1.5
2
B
OR
0.05
0.2
1
5
20
Subgroups
>12 months DAPT treatment vs. <12 months DAPT treatment
12 months DAPT treatment vs. <12 months DAPT treatment

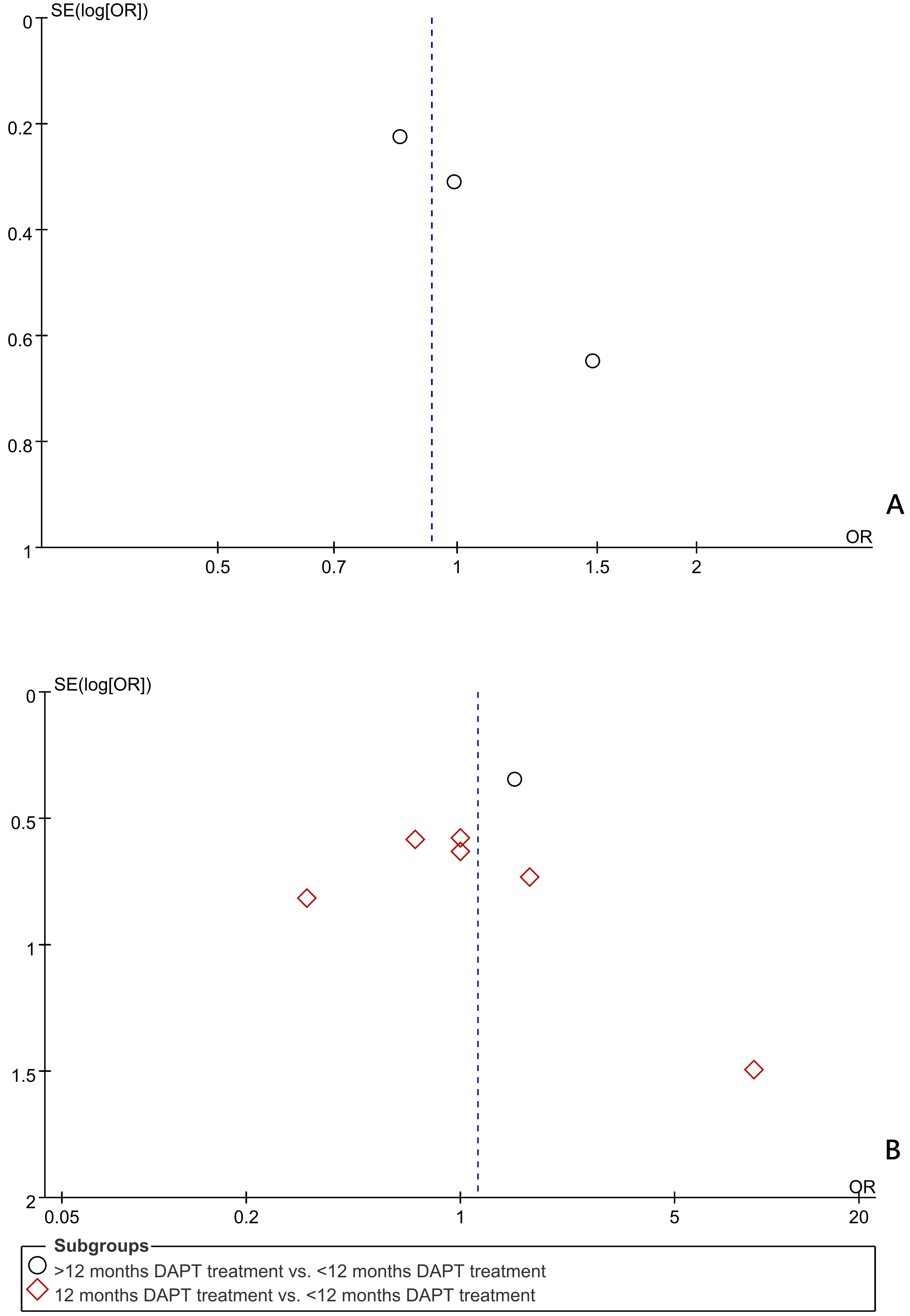
SE(log[OR])
0
0.2
0.4
0.6
0.8
1
OR
0.5
0.7
1
1.5
2
A
SE(log[OR])
0
0.5
1
1.5
2
OR
0.05
0.2
1
5
20
B
Subgroups
>12 months DAPT treatment vs. <12 months DAPT treatment
12 months DAPT treatment vs. <12 months DAPT treatment

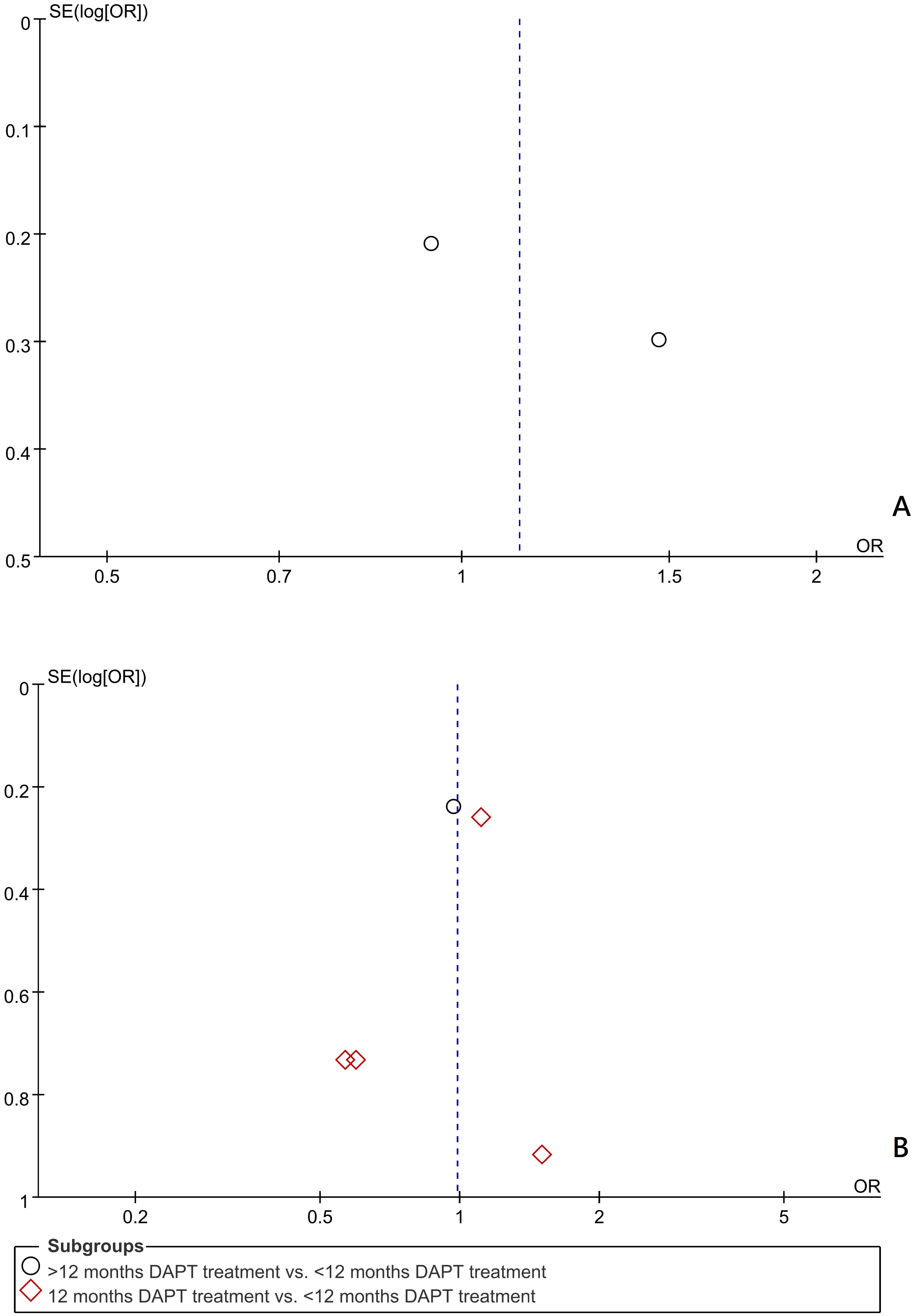
SE(log[OR])
0
0.1
0.2
0.3
0.4
0.5
OR
0.5
0.7
1
1.5
2
A
SE(log[OR])
0
0.2
0.4
0.6
0.8
1
OR
0.2
0.5
1
2
5
B
Subgroups
>12 months DAPT treatment vs. <12 months DAPT treatment
12 months DAPT treatment vs. <12 months DAPT treatment

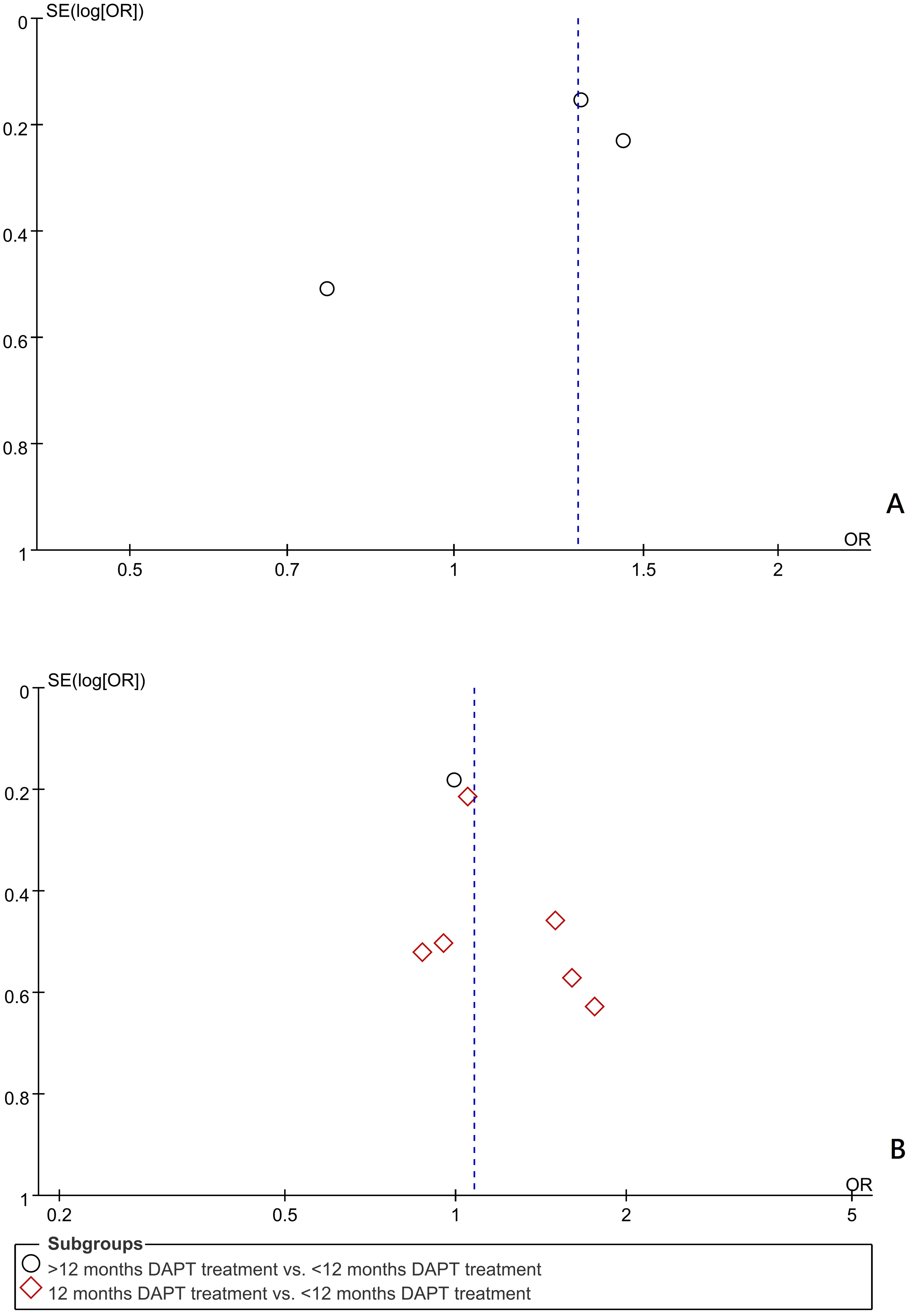
SE(log[OR])
0
0.2
0.4
0.6
0.8
1
OR
0.5
0.7
1
1.5
2
A
SE(log[OR])
0
0.2
0.4
0.6
0.8
1
OR
0.2
0.5
1
2
5
B
Subgroups
>12 months DAPT treatment vs. <12 months DAPT treatment
12 months DAPT treatment vs. <12 months DAPT treatment

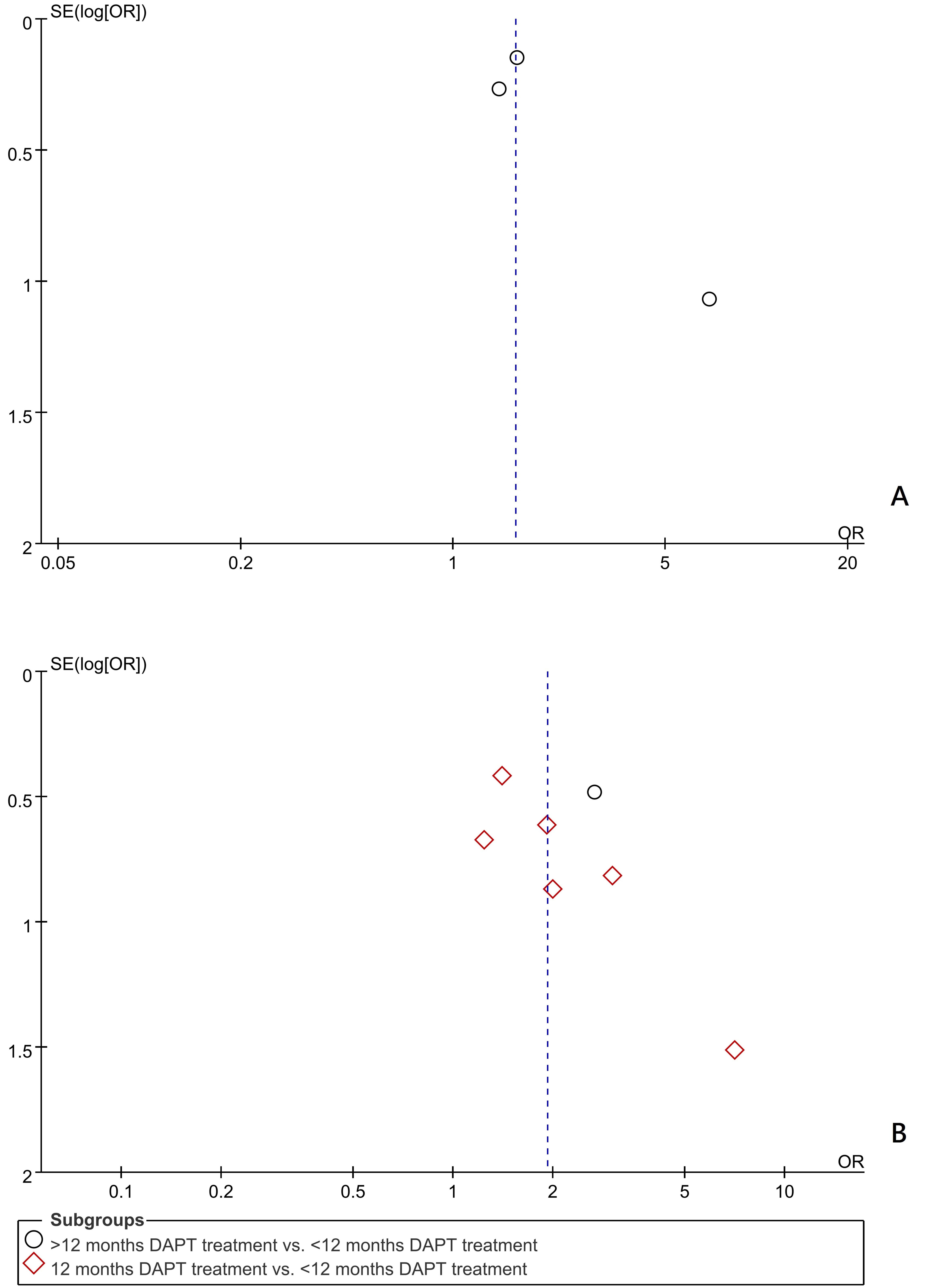
SE(log[OR])
0
0.5
1
1.5
2
0.05
0.2
1
5
20
OR
A
SE(log[OR])
0
0.5
1
1.5
2
0.1
0.2
0.5
1
2
5
10
OR
B
Subgroups
>12 months DAPT treatment vs. <12 months DAPT treatment
12 months DAPT treatment vs. <12 months DAPT treatment
